# Comparison of Yellow and Blue Sticky Cards for Detection and Monitoring Parasitoid Wasps of the Invasive *Halyomorpha halys* (Hemiptera: Pentatomidae)

**DOI:** 10.1093/jisesa/ieab062

**Published:** 2021-09-02

**Authors:** Mark Cody Holthouse, Lori R Spears, Diane G Alston

**Affiliations:** Biology Department, Utah State University, 5305 Old Main Hill, Logan, UT 84322, USA

**Keywords:** *Anastatus* spp, bycatch, parasitoid, survey, *Telenomus* spp, *Trissolcus* spp

## Abstract

The invasive *Halyomorpha halys* (Stål) is a significant agricultural and urban nuisance pest in many parts of the world. In North America, biological control of *H. halys* by parasitoid wasps in the families Scelionidae and Eupelmidae has shown promise. An effective technique for detection and monitoring native and exotic parasitoids is the deployment of yellow sticky cards; however, yellow cards also attract nontarget arthropods, reducing efficiency and accuracy of parasitoid screening. This study sought to identify an alternative yet effective trapping technique by comparing the number of target parasitoid wasps [Anastatus spp. Motschulsky (Hymenoptera: Eupelmidae), Telenomus spp. Haliday (Hymenoptera: Scelionidae), and Trissolcus spp. Ashmead (Hymenoptera: Scelionidae)] and arthropod bycatch on yellow and blue sticky cards deployed in urban, orchard, and vegetable landscapes in northern Utah from late May to early October in 2019 and 2020. Yellow sticky cards captured 54–72% more target parasitoids than blue cards from June through August in all three landscape types in both years; however, a positive correlation in parasitoid capture indicated blue cards detect target parasitoids, just in fewer numbers. Both card colors detected adventive *Trissolcus japonicus* (Ashmead) (Hymenoptera: Scelionidae) in initial findings of 2019, and in expanded locations of 2020. Furthermore, blue cards captured 31–48% less Diptera and nontarget Hymenoptera than yellow cards in both years across all three landscapes, translating to reduced card processing time and impacts to beneficial insect populations. Our results suggest that blue vs yellow sticky cards offer an alternative monitoring technique to survey for *H. halys* parasitoids.

During the past two decades, *Halyomorpha halys* (Stål) has become a significant agricultural and urban nuisance pest in North America (Hoebeke and Carter 2003, Leskey et al. 2012, Rice et al. 2014, Lee 2015) as well as in many other parts of the world (Haye et al. 2015). Consequently, much research has focused on assessment of biological control agents available in its native range of Asia that offer effective population suppression, specifically parasitoid wasps (Yang et al. 2009, Lee et al. 2013). There has been particular emphasis on *Trissolcus japonicus* (Ashmead) (Hymenoptera: Scelionidae), due to its efficiency in parasitizing *H. halys* eggs in its native range (Talamas et al. 2013, Zhang et al. 2017), and because adventive populations have been detected in North America (Talamas et al. 2015a, Milnes et al. 2016, Jarrett et al. 2019, Holthouse et al. 2020).

Monitoring efforts for the exotic *T. japonicus* and native parasitoid wasp species capable of parasitizing *H. halys* eggs have been conducted across North America and Europe using egg mass surveys (Herlihy et al. 2016, Abram et al. 2017, Dieckhoff et al. 2017, Stahl et al. 2019a, Zapponi et al. 2020) and yellow sticky cards (Lowenstein et al. 2019, Schumm et al. 2019, Peterson et al. 2021). Though both techniques are capable of parasitoid detection, yellow cards offer a more convenient approach to long-term and large-scale parasitoid detection and monitoring efforts (Quinn et al. 2019). The color yellow has been proven to attract some Scelionidae parasitoid wasp species (Ferreira Santos de Aquino et al. 2012), and sticky cards remain effective at detecting parasitoids in the field for longer durations than stink bug egg masses, as eggs may experience predation or decreased parasitoid attraction within just a few days (e.g., fresh eggs) (Qiu et al. 2007, Morrison et al. 2016). One downside to the convenience of yellow card deployment is the attraction and inadvertent capture of bycatch, or nontarget arthropods (Yee et al. 2013a, 2013b, Horton et al. 2019). Bycatch is known to impede or slow the processing time of target taxa, increase trap processing costs, and can result in the undesired mortality of rare and beneficial insects (Weber and Ferro 1991, Cha et al. 2015, Spears and Ramirez 2015, Jorgensen et al. 2020). Many of the target Nearctic Scelionidae that parasitize *H. halys* eggs are 1–2 mm in length (Talamas et al. 2015b) and can be easily concealed or damaged by bycatch of larger arthropod taxa.

Exploring alternative trap lures, sizes, shapes, colors, and other features is critical to mitigation of bycatch, while facilitating accurate monitoring of target species (Yee 2013a, Spears et al. 2016, Grocock et al. 2020). It is known that Hymenoptera are most sensitive to light reflectance in the UV, blue, green, and yellow spectrums (Peitsch et al. 1992, Briscoe and Chittka 2001, Ferreira Santos de Aquino et al. 2012). The current study sets out to compare efficacy of blue and yellow sticky cards, assess bycatch rates, and develop alternative options for parasitoid detection and survey in diverse landscapes (urban, orchard, and vegetable) with known *H. halys* populations in northern Utah.

## Materials and Methods

### Card Deployment

Double-sided yellow and blue sticky cards (20 × 14 cm; Alpha Scents Inc., West Linn, OR) were deployed for 14 d (±1 d) intervals beginning 29–30 and 25–28 May in 2019 and 2020 respectively, and ending on 1 October in both years, resulting in nine deployment periods in each year. Cards were deployed at 24 sites in Cache, Davis, Salt Lake, Utah, and Weber counties of northern Utah. Sites represented eight replications of three landscape types: residential urban, vegetable row crops, and fruit orchards. At each site, one yellow and one blue card were placed at approximately 2 m height and 1 m apart. Cards were attached to tree branches or metal stakes using twist ties. Manufacturer wavelength reflectance specifications for the yellow and blue cards were 575.12 nm and 466.19 nm, respectively. After collection, biodegradable plastic straws (Evriholder, Anaheim, CA) were placed as spacers between cards to prevent adherence, and stored at –13°C until processing. The duration of card storage was variable, from a few days to several months.

### Card Processing

Each card was examined under a stereomicroscope (Leica Stereozoom S9E, Leica Microsystems Inc.) with 97.6x–880x magnification for the presence of target parasitoid genera, which included *Anastatus* spp. (Hymenoptera: Eupelmidae), *Telenomus* spp. (Hymenoptera: Scelionidae), and *Trissolcus* spp. (Hymenoptera: Scelionidae). These genera were selected because they have been observed parasitizing *H. halys* eggs in Utah (Holthouse et al. 2020) and elsewhere in the U.S. (Talamas et al. 2015a, Abram et al. 2017, Dieckhoff et al. 2017, Stahl et al. 2019b). Target wasps were removed from cards with a 10 mm diameter cork borer/punch (Cole-Parmer, Vernon Hills, IL) to support convenient manipulation of specimens on small pieces of card stock. Wasps encased in the viscous adhesive were cleaned by soaking in Histo-Clear II histological clearing fluid (National Diagnostics, Atlanta, GA) for ~5–7 min. After drying, wasps were point-mounted to an insect pin for identification. Wasps in the family Scelionidae were identified to genus using keys by Johnson (1984), Masner (1980), and Talamas (2015b); wasps in the family Eupelmidae were identified using an annotated key by Gibson et al. (1997) and a key on North American *Anastatus* spp. by Burks (1967). Species-level identification was only performed for *Trissolcus* spp. (Talamas 2015b) since surveys were focused on detecting the exotic *T. japonicus*. Some *Trissolcus* specimens were unidentifiable beyond genus level due to physical damage. All intact parasitoid wasps were pinned, labeled, and stored in the Alston Lab, Department of Biology, Utah State University, Logan, UT. Voucher specimens were deposited in the Utah State University Insect Collection.

The number of bycatch arthropods per card was recorded by order. Counts were capped at 100 individuals per order to support sustainable processing time. The time required to count bycatch was recorded for a subgroup of 66 blue and 64 yellow sticky cards deployed between late August and early September 2020; a time period associated with moderate capture of target parasitoids and bycatch. Processing times to remove and identify target parasitoids were not included in this analysis.

### Statistical Analyses

Linear mixed-effects models were used to test the main and interaction effects of card color, landscape type, and deployment period for the mean number of target wasps and mean number of combined Diptera and nontarget Hymenoptera per card. Square root and log transformations were used for the target wasp and bycatch models, respectively, and to meet model normality distribution assumptions. Contrasts using Tukey’s HSD method, adjusted for type one error, were implemented for posthoc analysis of pairwise mean comparisons. For all tests, *α* was set to 0.05. Spearman’s correlation coefficient tests between numbers of target wasp captures on yellow and blue sticky cards were conducted for each year and individual landscape type. All models were conducted using various functions from packages *lme4* (Bates et al. 2015), *car* (Fox and Weisberg 2019), *emmeans* (Lenth 2020), and *ggpubr* (Kassambara 2020) within R software (R version 4.0.2; R Core Team 2020). All figures were created using the ggplot2 package in R (Wickham 2016).

Deployment period 9, representing the last deployment date in late September, was removed from both 2019 and 2020 analyses because the mean number of wasps was at or near zero for all sites due to adverse weather conditions. One vegetable site in Salt Lake City was removed from 2019 analyses due to extremely high numbers of parasitoid wasps in the first two and very last deployment periods that year. This vegetable site was in close proximity to residential homes, contained a high diversity of plant species, and was surrounded by several fruit trees; these factors likely contributed to the abnormally high number of parasitoid wasps found there. Excluding this site allowed for improved homogeneity of variance and normality of residuals in these models. Due to high winds and vandalism, four sticky cards were lost before collection in 2019 and eight in 2020 out of a total of 368 and 384 analyzed cards per year, respectively; these cards were excluded from statistical analyses.

## Results

### Target Parasitoids

In both years of the survey, there were approx. three times more target parasitoid wasps detected on yellow than blue sticky cards: 1,608 vs 560 in 2019, and 966 vs 301 in 2020. The majority of captured parasitoids belonged to the genus *Trissolcus* (72–88%), followed by *Telenomus* (11–28%). For both card colors, *Trissolcus* spp. were captured most frequently in urban landscapes, whereas *Telenomus* spp. were more common in vegetable sites. *Anastatus* spp. accounted for ≤ 2% of target wasps detected on all cards across all landscape types and years (Table 1).

**Table 1.** The number of target parasitoid wasps by genus captured on blue and yellow sticky cards in urban, orchard, and vegetable landscapes in 2019 and 2020 combined

Color	Landscape	Anastatus	Telenomus	Trissolcus	Total
Blue	Urban	2 (0.01)	53 (0.15)	300 (0.85)	355
	Orchard	1 (0.00)	65 (0.28)	169 (0.72)	235
	Vegetable	5 (0.02)	62 (0.23)	204 (0.75)	271
Yellow	Urban	5 (0.00)	129 (0.11)	1,007 (0.88)	1,141
	Orchard	3 (0.00)	127 (0.15)	710 (0.85)	840
	Vegetable	0	145 (0.24)	448 (0.76)	593

Proportional abundance of each genus by card color and landscape type is shown in parentheses.

The vast majority of *Trissolcus* spp. captured were *Trissolcus euschisti* (Ashmead), primarily in urban and orchard landscapes for both blue (63–66% of all *Trissolcus* captured) and yellow (65–67% of *Trissolcus*) cards. Capture of *T. japonicus* occurred most commonly in urban and orchard landscapes, but accounted for ≤ 9% of the total *Trissolcus* detected for any one card color and landscape type. *Trissolcus hullensis* (Harrington) was found most commonly within vegetable and orchard landscapes on blue (3–15% of *Trissolcus*) and yellow (7–10% of *Trissolcus*) cards. *Trissolcus utahensis* (Ashmead) occurred most commonly in vegetable landscapes on blue (18% of *Trissolcus*) and yellow (25% of *Trissolcus*) cards. There was a total of 648 unidentifiable *Trissolcus* spp. due to physical damage; approx. 1/4 of the captured wasps in all landscape types for blue (27–29%) and yellow (19–25%) cards (Table 2).

**Table 2.** The number of *Trissolcus* spp. identified on blue and yellow sticky cards in urban, orchard, and vegetable landscapes in 2019 and 2020 combined

Color	Landscape	T. japonicus	T. euschisti	T. hullensis	T. utahensis	Trissolcus spp.	Total
Blue	Urban	10 (0.03)	199 (0.66)	5 (0.02)	5 (0.02)	81 (0.27)	300
	Orchard	3 (0.02)	106 (0.63)	5 (0.03)	8 (0.05)	47 (0.28)	169
	Vegetable	2 (0.01)	75 (0.37)	31 (0.15)	37 (0.18)	59 (0.29)	204
Yellow	Urban	87 (0.09)	679 (0.67)	6 (0.01)	24 (0.02)	211(0.21)	1,007
	Orchard	16 (0.02)	458 (0.65)	48 (0.07)	52 (0.07)	136 (0.19)	710
	Vegetable	4 (0.01)	174 (0.39)	46 (0.10)	110 (0.25)	114 (0.25)	448

*Trissolcus* wasps that were unidentifiable below the genus level due to damage or obstruction of view are presented as *Trissolcus* spp. Proportional abundance of each species by card color and landscape type is provided in parentheses.

**Fig. 1.**
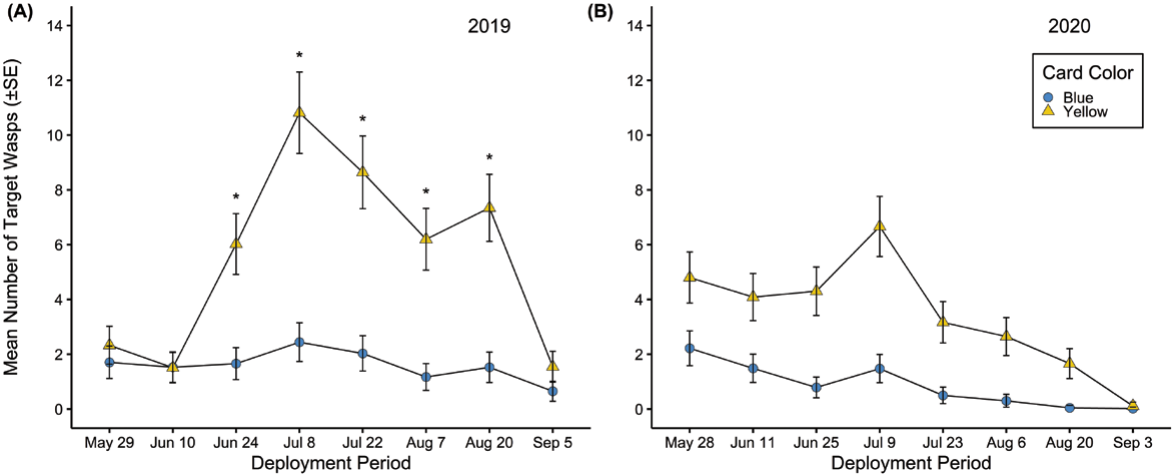
Interactions for card color (yellow and blue) by deployment period (14 ± 1-d intervals late May to September) for mean number of target parasitoid wasps (±SE) in 2019 (A) (*P* < 0.001) and 2020 (B) (*P* = 0.06). * represent significant differences between yellow and blue sticky card mean wasp capture within a deployment period (Tukey HSD; *P* < 0.05). The interaction between color and deployment was not significant in 2020.

**Fig. 2.**
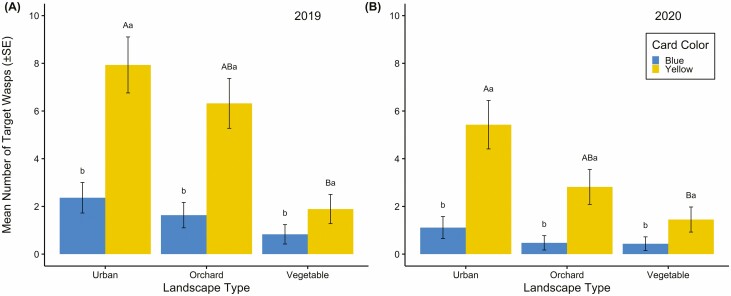
Interactions for card color (yellow and blue) by landscape type (urban, orchard, and vegetable) for mean number of target parasitoid wasps (±SE) captured from May to September 2019 (A) and 2020 (B). Lowercase letters represent significant differences between card colors within a landscape type. Uppercase letters represent significant differences in parasitoid capture among landscapes for yellow cards (Tukey HSD; *P* < 0.05). There were no differences in parasitoid detection on blue cards among landscape types (*P* > 0.05).

Higher numbers of target parasitoids were captured on yellow compared to blue cards from late June through August in 2019 (Fig. 1A) resulting in a significant card color by deployment period interaction (Table 3). In contrast, in 2020, this interaction was not significant (Table 3); however, that year more parasitoids were captured on sticky cards deployed in late May through early July (Fig. 1B; significant main effect for deployment, Table 3). Further, the number of parasitoids on yellow cards was greater than blue in urban, orchard, and vegetable landscapes in both years (Table 3, Fig. 2A and B). Yellow cards in urban landscapes captured more parasitoids than yellow cards in vegetable landscapes in both years (Fig. 2A and B). In 2019, parasitoid captures on yellow cards in orchards were also greater than those in vegetable landscapes (Fig. 2A). The mean number of parasitoids captured by blue cards did not differ among landscape types (Fig. 2A and B). Overall, more target parasitoids were captured in 2019 than 2020 (Figs. 1 and 2).

**Table 3.** Results from the linear mixed effects model for mean target parasitoids by sticky card color, landscape, deployment period, and subsequent interactions in 2019 and 2020

Year	Treatment	F	df	P
2019	Color	95.96	1, 300	<0.001*
	Landscape	7.29	2, 20	0.004*
	Deployment	11.23	7, 300	<0.001*
	Color × Landscape	6.55	2, 300	0.002*
	Color × Deployment	5.48	7, 300	<0.001*
	Landscape × Deployment	1.29	14, 300	0.21
	Color × Landscape × Deployment	1.51	14, 300	0.10
2020	Color	104.86	1, 315	<0.001*
	Landscape	3.63	2, 21	0.04*
	Deployment	19.36	7, 315	<0.001*
	Color × Landscape	5.40	2, 315	0.01*
	Color × Deployment	1.95	7, 315	0.06
	Landscape × Deployment	1.64	14, 315	0.07
	Color × Landscape × Deployment	0.60	14, 315	0.87

*indicates a significant difference amongst treatment group means (*P* < 0.05).

Target parasitoid captures on yellow cards were positively correlated with captures on blue cards in 2019 (*r* = 0.43, df = 182 *P* < 0.001) and 2020 (*r* = 0.62, df = 190, *P* < 0.001). In 2019, there was a positive correlation between parasitoid captures on blue and yellow cards in urban (*r* = 0.42, df = 62, *P* < 0.001) and orchard (*r* = 0.31, df = 62, *P* = 0.01) landscapes, but not for vegetable (*r* = 0.23, df = 54, *P* = 0.08) landscapes. However, in 2020, there was a correlation in parasitoid captures on blue and yellow cards within all three landscape types (urban: *r* = 0.67, df = 62, *P* < 0.001; orchard: *r* = 0.49, df = 62, *P* < 0.001; vegetable: *r* = 0.62, df = 62, *P* < 0.001).

### Bycatch

There were 1.8 and 1.6 times more total bycatch individuals caught on yellow than blue cards in 2019 (45,844 vs 25,920) and 2020 (48,594 vs 30,888). The highest bycatch captures occurred in vegetable landscapes for both card colors (1.5–2.6 and 1.3–1.9 times more in vegetable than orchard and urban for blue and yellow, respectively). The vast majority of bycatch belonged to the Orders Diptera, Thysanoptera, Hymenoptera, and Hemiptera. Other bycatch was in the Orders Araneae, Coleoptera, Dermaptera, Ephemeroptera, Lepidoptera, Neuroptera, and Odonata. The mean assessment time per card required to count bycatch individuals was 3 min and 54 s for blue cards and 5 min and 40 s for yellow cards (assessment times measured in late August and early September 2020, only), a 1.5 times increase in processing time for yellow cards.

Diptera were the most abundant bycatch taxa in urban and orchard landscapes accounting for 50% and 38% of the bycatch, respectively, on blue cards, and for 42–48% on yellow cards. Thysanoptera were the most abundant bycatch order on blue cards in vegetable landscapes (41% of the bycatch), with Diptera and Thysanoptera each accounting for 31% of bycatch on yellow cards in vegetable landscapes.

**Fig. 3.**
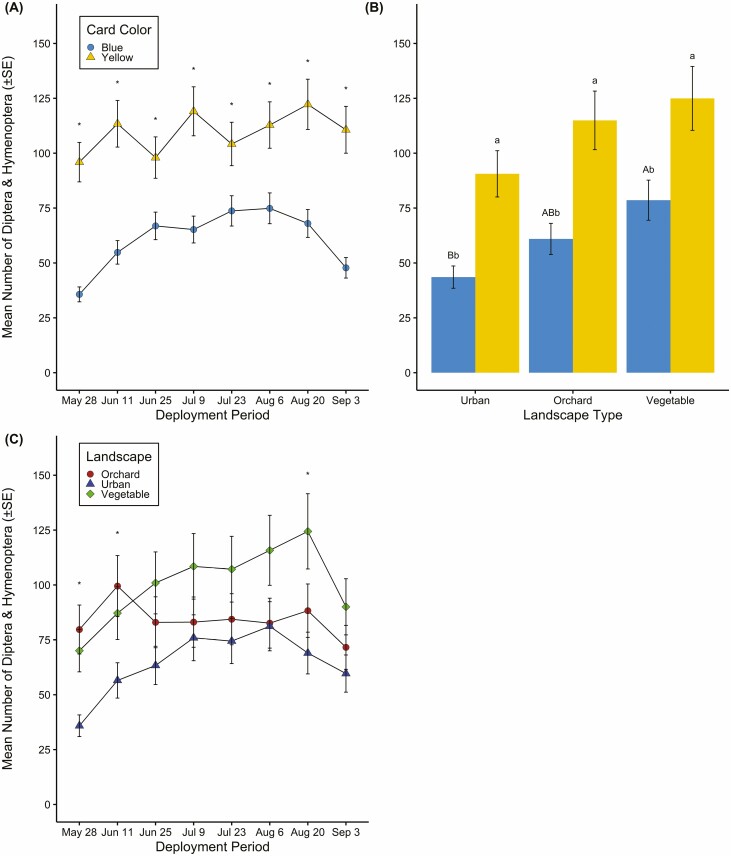
(A) Interactions for card color and deployment period (14 ± 1-d intervals late May to September) for the mean number of Diptera and nontarget Hymenoptera (±SE) bycatch in 2020. * represent significant differences between yellow and blue sticky card mean capture within a deployment period. (B) Card color by landscape type (urban, orchard, and vegetable) interactions for mean number of Diptera and nontarget Hymenoptera (±SE) captured from May to September 2020. Lowercase letters represent significant differences between card colors within a landscape type. Uppercase letters represent significant differences in bycatch capture among landscapes for blue cards. There were no differences in bycatch on yellow cards among landscape types (*P* > 0.05). (C) Landscape type by deployment period interaction for the mean number of Diptera and nontarget Hymenoptera (± SE) in 2020. * represent significant differences between landscape mean captures within a deployment period. (Tukey HSD; *P* < 0.05).

Results from the linear mixed-effects model considering the mean number of Diptera and nontarget Hymenoptera revealed no interactions among card color, landscape type, or deployment in 2019; yet significant main effects revealed that more Diptera and nontarget Hymenoptera were captured by yellow than blue cards, and sticky cards deployed mid-season, specifically late June through late August, captured more Diptera and nontarget Hymenoptera (Tables 4 and 5). However, in 2020, all two-way interactions were significant (Table 4). More Diptera and nontarget Hymenoptera were caught on yellow compared to blue cards across all deployment periods (Table 4, Fig. 3A). Further, yellow cards captured significantly more Diptera and nontarget Hymenoptera than blue within all landscapes, and blue cards captured fewer bycatch in urban compared to vegetable landscapes; however, bycatch numbers on blue cards were similar in urban and orchard landscapes (Table 4, Fig. 3B). Diptera and nontarget Hymenoptera captures were highest in vegetable and orchard landscapes in the early part of the season, and in vegetable landscapes later in the season; urban landscapes captured the fewest Diptera and nontarget Hymenoptera across almost all deployment periods (Table 4, Fig. 3C).

**Table 4.** Results from the linear mixed effects model of mean Diptera and nontarget *Hymenoptera* by sticky card color, landscape type, deployment period, and subsequent interactions in 2019 and 2020

Year	Treatment	F	df	P
2019	Color	232.44	1, 296	<0.001*
	Landscape	2.81	2, 20	0.08
	Deployment	9.32	7, 296	<0.001*
	Color × Landscape	0.41	2, 296	0.67
	Color × Deployment	0.86	7, 296	0.54
	Landscape × Deployment	1.66	14, 296	0.06
	Color × Landscape × Deployment	0.30	14, 296	0.99
2020	Color	0	1, 303	<0.001*
	Landscape	0	2, 303	0.03*
	Deployment	0	7, 303	<0.001*
	Color × Landscape	0	2, 303	0.01*
	Color × Deployment	0	7, 303	<0.001*
	Landscape × Deployment	0	14, 303	0.001*
	Color × Landscape × Deployment	0	14, 303	0.48

* indicates a significant difference amongst treatment group means (*P* < 0.05).

**Table 5.** Mean (x̄) and standard error (±SE) of Diptera and nontarget Hymenoptera captured in 2019 listed by color and deployment period

Independent Variables	$\bar{x}$ (± SE)
*Color*	
Blue	56.6 (4.24)^b^
Yellow	110.3 (8.28)^a^
*Deployment (Date)*	
1 (29 May)	59.0 (5.49)^d^
2 (10 June)	70.0 (6.43)^bcd^
3 (24 June)	78.6 (7.27)^bc^
4 (8 July)	89.4 (8.21)^ab^
5 (22 July)	106.0 (9.74)^a^
6 (7 Aug)	82.7 (7.60)^abc^
7 (20 Aug)	90.1 (8.28)^ab^
8 (5 Sept)	66.3 (6.14)^cd^

Letters represent significant differences between mean bycatch capture between colors and deployment periods (Tukey HSD; *P* < 0.05).

## Discussion

Both yellow and blue sticky cards detected a similar complex of target parasitoid wasps; however, yellow cards captured significantly higher numbers compared to blue cards across all landscape types and during most deployment periods. Both yellow and blue cards effectively detected peaks in wasp abundance in July and early August, likely correlated with warmer temperatures and increased stink bug host (e.g., *H. halys*) egg abundance (Nielsen et al. 2016, Holthouse et al. 2021). The relatively low target wasp capture in late May to early June of 2019, as well as early September of both years was likely the result of lower temperatures (mean minimum daily temperature May 25 to June 10 in Salt Lake City, Utah: 2019 = 8.94°C, 2020 = 12.17°C) or shorter photoperiods which can result in diminished wasp development and activity (Arakawa and Namura 2002, Hance et al. 2007, Stahl et al. 2019b, Cordeiro and Bueno 2021, climate.usu.edu).

The majority of target wasps captured on sticky cards belonged to the genus *Trissolcus*, specifically the native species *T*. *euschisti*. This species has been documented in previous parasitoid surveys in northern Utah (Schumm et al. 2019, 2020b), and is known to successfully complete development and emerge from *H. halys* eggs (Abram et al. 2019, Jarrett et al. 2019, Holthouse et al. 2020). *T. euschisti* and *T. japonicus* were most commonly detected in residential urban, followed by orchard landscapes. Both species prefer arboreal habitats where their primary stink bug hosts occur (Okuda and Yeargan 1988, Talamas et al. 2015a, Cornelius et al. 2016, Jones et al. 2017, Kaser et al. 2018, Peterson et al. 2021, Quinn et al. 2021). *T. hullensis* and *T. utahensis* were detected most commonly in vegetable landscapes, where their stink bug hosts (e.g., *Euschistus* spp.) are known to commonly feed and oviposit (Pease and Zalom 2010, Ganjisaffar et al. 2020).

Very importantly, the first detection of *T. japonicus* in Utah was documented in this study. *T. japonicus* was detected on both card colors in Salt Lake and Weber counties in 2019 (Holthouse et al. 2020), and expanded to Utah County in 2020. This study represents the first documentation of blue sticky cards being used to effectively monitor for *T. japonicus*.

Both card colors captured a large proportion of unidentifiable *Trissolcus* spp. due to physical damage caused by the sticky adhesive. These damaged specimens can result in false negatives of important parasitoid species. Fortunately, future sticky card monitoring and detection efforts will be less reliant on the morphological integrity of specimens for identification, as a new species-specific molecular identification technique for *T. japonicus* was recently created by Chen et al. (2021).

*Telenomus* was the second most commonly detected genus on both blue and yellow cards, indicating that these parasitoids are either less attracted to yellow and blue cards than *Trissolcus* or have smaller wild populations in the areas surveyed. Other similar sticky card surveys have documented higher numbers of *Telenomus* over *Trissolcus* species on yellow cards, indicating the observed density of genera in this study may be regionally specific (Peterson et al. 2021). The abundance of *Telenomus* varied across landscapes, but orchard and vegetable landscapes had proportionally higher detections, as noted in other research on *Telenomus podisis* Ashmead (Okuda and Yeargan 1988, Ogburn et al. 2016). This species can successfully parasitize *H. halys* eggs, but usually at low levels (Dieckhoff et al. 2017, Holthouse et al. 2020), and has been documented on yellow cards in other *H. halys* parasitoid surveys (Quinn et al. 2021). We did not identify *Telenomus* to species and it is possible that captured individuals represent species incapable of stinging or successfully parasitizing *H. halys* (Peterson et al. 2021).

Similar to the results of Quinn et al. (2021), relatively few *Anastatus* were detected on cards in this study, with no apparent card color or landscape type preference. Based on the low number of individuals captured in this genus, wild and sentinel egg mass surveys may be more effective than sticky cards at detecting species of *Anastatus* capable of attacking *H. halys* eggs (Jones et al. 2014, 2017, Dieckhoff et al. 2017).

**Fig. 4.**
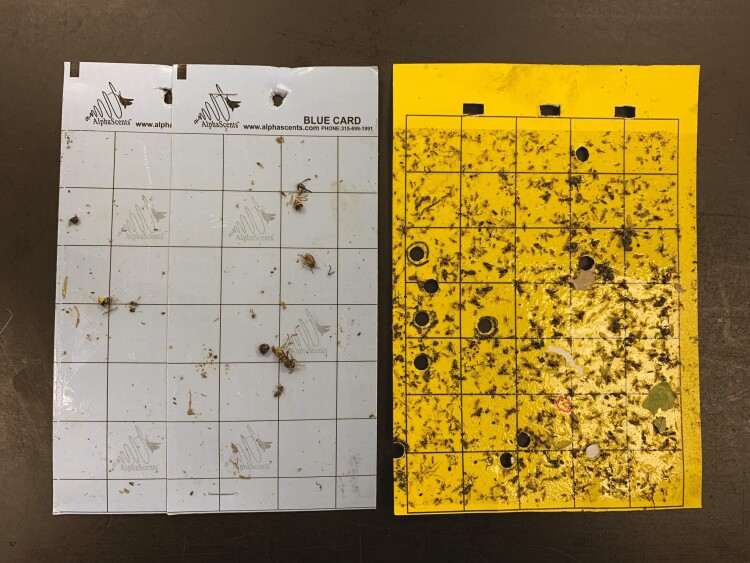
Blue (left) and yellow (right) sticky cards collected from the same site 14 d after deployment. Black circles on the yellow card represent locations where target parasitoids were removed from the card in order for species identification.

Although valuable information can be gained through examination of bycatch (Buchholz et al. 2011, Spears and Ramirez 2015), Diptera and nontarget Hymenoptera captures contributed to greater processing times, especially for yellow cards where bycatch captures were 1.5–2 times greater than on blue cards. Numerous bycatch individuals were similar in size, morphology, or color to the target parasitoid wasps. Diptera regularly covered large portions of the sticky card surface, sometimes obscuring other specimens (Fig. 4). Though Thysanoptera and Hemiptera were also captured in high numbers, they were generally smaller or easier to distinguish from target parasitoid wasps and therefore not considered as disruptive to card processing. Because of this, bycatch analyses were focused on combined Diptera and nontarget Hymenoptera captures. These insects were captured in high abundances from June through August when target parasitoids were also at their peak abundance; this observation was also noted by Quinn et al. (2021). The higher captures of Diptera and nontarget Hymenoptera during this period may outweigh the benefit of increased captures of target parasitoids for yellow cards. In contrast, blue cards also detected target parasitoids during this peak activity time, but with substantially reduced bycatch. Further, monitoring efforts hoping to capitalize on higher *T. japonicus* detections in July and August may want to target residential urban and orchard landscapes, which capture less Diptera and nontarget Hymenoptera during this peak time period compared to vegetable landscapes. It is also important to note that this study only accounted for the first 100 specimens per order encountered on each card, which means our results underestimate bycatch, especially for Diptera and Thysanoptera which often exceeded this upper limit.

Insects in the Order Hymenoptera provide important ecosystem services (Noriega et al. 2018). Many are pollinators (Southwick and Southwick 1992, Klein et al. 2007), some of which are experiencing population declines due to habitat loss, pathogens and pests, improper management practices, and other anthropogenic factors (Watanabe 1994, Colla and Packer 2008, Brown and Paxton 2009, Potts et al. 2016). It is unknown if sticky cards impact nontarget Hymenoptera populations; however, we demonstrated that the use of blue vs yellow cards would reduce nontarget captures, as was similarly noted by Atakan et al. (2016). Interestingly, blue cards in 2020 caught almost as many Hymenoptera as yellow cards in vegetable landscapes, likely due to the greater diversity of angiosperm plant species present in these landscapes (Hülsmann et al. 2015, Lanner et al. 2020). However, some Hymenoptera, such as *Bombus* spp., are highly attracted to the color blue (Stephen and Rao 2005), so blue cards may need to be avoided in vegetable landscapes.

The current study only addresses the disparity between target parasitoid and bycatch captures on yellow and blue sticky cards. This difference may have been due to the use of a suboptimal blue card color or other physical properties. Work done by Yee (2013a, 2015) found that different color shades, transparency properties, and direction of ambient lighting of yellow traps can elicit varying levels of response in the fruit fly *Rhagoletis indifferens* Curran, and this may also apply to different blue colors as well. Future studies may therefore seek to compare other traps with different properties to determine the most effective approach for attracting target parasitoids and minimizing bycatch. Different types of sticky card adhesives have demonstrated differing levels of effectiveness in insect capture, but both blue and yellow sticky cards in this study contained the same type of adhesive, so this is likely not a factor in these observed differences (Kaloostian 1961, Davidson et al. 2015).

Although relatively unselective trap types like yellow cards are sometimes necessary for novel or adventive insect monitoring programs where only a few potential target organisms are present (Marchioro et al. 2020), future *H. halys* parasitoid monitoring efforts may look to adopt slightly less attractive card colors in the pursuit of decreased bycatch or target species specificity. Blue cards offer low, but consistent season-long captures of target parasitoids in urban and orchard landscapes. Blue cards documented similar seasonal parasitoid population trends as yellow cards, similar to a study that compared efficacy of different trap types for *H. halys* (Acebes-Doria et al. 2018, 2020). As compared to yellow cards, blue cards enable faster processing times and reduced impact on beneficial insect populations. Importantly, blue cards successfully documented the first detection of *T. japonicus* in northern Utah, which is a primary target of *H. halys* biological control surveys in North America (Peterson et al. 2021, Quinn et al. 2021). Since blue cards have faster processing times, producers and pest managers can deploy more cards, allowing for greater spatial and temporal coverage. More testing is necessary to fully appreciate the practical use of blue cards in monitoring for parasitoids of *H. halys*, but in light of the nearly global pest status of this invasive insect, this study offers the first look into an alternative sticky card monitoring technique.
